# Recent Advances in Modified Cellulose for Tissue Culture Applications

**DOI:** 10.3390/molecules23030654

**Published:** 2018-03-14

**Authors:** James C. Courtenay, Ram I. Sharma, Janet L. Scott

**Affiliations:** 1 Centre for Sustainable Chemical Technologies, University of Bath, Bath BA2 7AY, UK; R.Sharma@bath.ac.uk (R.I.S.); J.L.Scott@bath.ac.uk (J.L.S.); 2 Department of Chemistry, University of Bath, Bath BA2 7AY, UK; 3 Department of Chemical Engineering, University of Bath, Bath BA2 7AY, UK

**Keywords:** tissue engineering, sustainable chemistry, cellulose, biomaterials, surface modifications, cell culturing, regenerative medicine

## Abstract

Tissue engineering is a rapidly advancing field in regenerative medicine, with much research directed towards the production of new biomaterial scaffolds with tailored properties to generate functional tissue for specific applications. Recently, principles of sustainability, eco-efficiency and green chemistry have begun to guide the development of a new generation of materials, such as cellulose, as an alternative to conventional polymers based on conversion of fossil carbon (e.g., oil) and finding technologies to reduce the use of animal and human derived biomolecules (e.g., foetal bovine serum). Much of this focus on cellulose is due to it possessing the necessary properties for tissue engineering scaffolds, including biocompatibility, and the relative ease with which its characteristics can be tuned through chemical modification to adjust mechanical properties and to introduce various surface modifications. In addition, the sustainability of producing and manufacturing materials from cellulose, as well as its modest cost, makes cellulose an economically viable feedstock. This review focusses specifically on the use of modified cellulose materials for tissue culturing applications. We will investigate recent techniques used to promote scaffold function through physical, biochemical and chemical scaffold modifications, and describe how these have been utilised to reduce reliance on the addition of matrix ligands such as foetal bovine serum.

## 1. Introduction

Organ failure is one of the most frequent, devastating and costly problems in human healthcare. Tissue engineering is an interdisciplinary field, enlisting expertise from engineering and life sciences towards the development of new biological substituents, through the regeneration of human cells, tissues or organs, in order to repair or replace and restore function to damaged tissue or organs [1,2]. This desire to heal those ill or wounded is a concept recounted in literature and religion throughout history [3], and pioneering practical research is now making tissue engineering a reality [4].

The first attempts to repair damaged organs often relied upon primitive biomaterials, such as ceramics, wood and metals used as implants or prosthetics [5]. Modern surgery and the scientific understanding of germ theory, sterilisation and anaesthesia, catalysed technical advancements leading to the introduction of skin grafts and reconstructive surgery founded in an understanding of cellular biology [3,4].

By the 20th century advances in science and medical practices made whole organ transplants feasible and the first human heart transplantation was conducted in 1967 by the South African surgeon Christian Bernard. Receiving much media interest at the time, it also sparked controversy over the ethical issues of transplantation. One major concern is that the host immune system might reject the transplant, thus voiding the purpose of the procedure [4].

Pioneering research by Green in 1977 investigated seeding a chondrocyte culture onto bone scaffolds and implanting these into mice to generate new cartilage [6,7]. Despite being unsuccessful, this work identified the process of culturing tissue by seeding cells onto an appropriate scaffold. Building on this, Burke and Constant, in 1982, attempted to generate a tissue engineered skin substitute using a collagen matrix to support the growth of dermal fibroblasts [8]. Others used sheets of keratinocytes to treat burn patents [9] and developed scaffolds from a collagen gel [10].

Limitations of using naturally sourced biomaterials (such as collagen) include their limited range of physical and chemical properties as well as source variability. To overcome these limitations, researchers turned to synthetic polymeric scaffold materials. The first synthetically produced polymeric scaffolds were used by Vacanti and Langer in 1993, who generated new tissue that could be implanted back into the body [11]. Their findings catapulted tissue engineering into the forefront of the public awareness after they published the image showing the now infamous “Auriculosaurus”—a mouse with a human ear. This demonstrated that tissue constructs could be further grown in vivo [11].

The first human to receive a tissue engineered implant was a young patient with Poland Syndrome in 1991. The implant was composed of a synthetic PLGA polymer scaffold seeded with chondrocytes and was intended to replace the patients absent sternum [7]. In 2008, the first transplantation of a tissue engineered trachea was conducted. This novel procedure used a decellularised trachea, from a human donor, which was seeded with cartilage cells derived from the patient’s own stem cells, as well as epithelial cells taken from a healthy part of their trachea [12]. Whether the scaffold functioned largely as a support for the airways, or actually induced regeneration of the epithelial lining within the tracheal implant, has been debated [13]. In 2014, to treat a patient with a severed spinal cord, surgeons seeded cells taken from the patient’s olfactory bulb onto strips of nerve fibres from the patient’s ankle, to form a bridge for the cells to grow across [14].

### 1.1. Principles of Tissue Engineering

**Figure 1:**
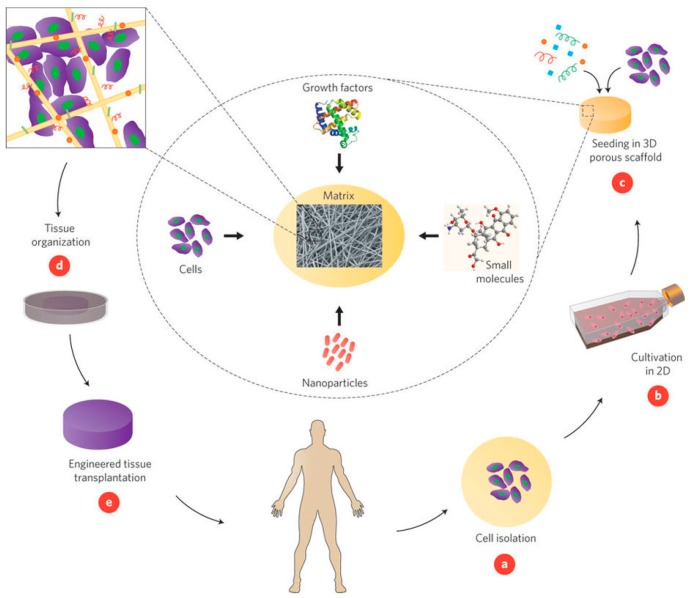
The key steps of tissue engineering: (a) cell isolation, (b) cultivation in 2D, (c) seeding in 3D porous scaffold, (d) tissue organisation and (e) engineering tissue transplantation. Figure reproduced from Dvir et al., 2011, Copyright © 2010, Springer Nature [16].

The basic principles of cell culture for tissue engineering commonly involve the use of living cells to repair or regrow tissue or an organ damaged by disease, or trauma, as described below and illustrated in Figure 1. The steps involved may include:

1. Desired cells are extracted from the patient;
2. The isolated cells are cultured and expanded in vitro on a 2D scaffold;
3. The cell culture is seeded into a 3D scaffold support and additional biomolecules, such as matrix ligands, are added to promote growth;
4. A bioreactor is often used to develop the cell/scaffold construct into functioning tissue; and
5. Once the functional tissue graft is generated, this is implanted onto the damaged site where it becomes integrated into the surrounding tissue, restoring tissue function [1,15].

To engineer tissue, there are traditionally three components: Cells, biomolecules, and a scaffold. Different cell lines can be used depending on where they are isolated from and the end application. Both allogenic and autologous cells can be used, but the later are obtained from the patient itself and therefore do not elicit an immune response from the recipient, thus mitigating the risk of implant rejection [15]. Stem cells may also be used as these can differentiate into various cell lines. Stem cells isolated from adult or embryonic tissues are the main types of human stem cells used for tissue engineering. Embryonic stem cells are pluripotent in nature, i.e., show unlimited proliferative capacity and potentially differentiate into all body cells, which is beneficial for culturing new tissue [17,18]. However, there are some ethical concerns associated with the use of embryonic stem cells, which are harvested from “excess” human embryos created for implantation following in vitro fertilisation. Adult stem cells are becoming more commonly used and are harvested from umbilical cord blood, bone marrow and even discarded fat tissue from liposuction procedures, which will reduce the need to use embryonic stem cells. However, the major limitations of adult stem cells are: (i) that they are multipotent, not pluripotent (there are fewer cell types that can be differentiated from adult stem cells than from embryonic stem cells) [19] and (ii) fewer population doublings occur in adult stem cells with fewer numbers of cell passages possible, leading to a slower doubling rate [20].

**Figure 2:**
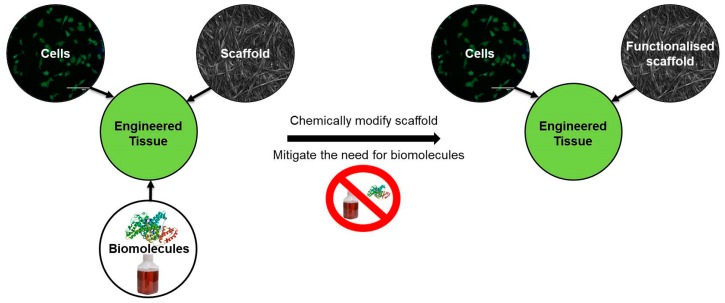
The traditional three-component system of tissue engineering vs. a two-component system, whereby the scaffolds have been functionalised to reduce the reliance on additional biomolecules such as FBS.

Biological molecules, including proteins, matrix ligands and growth factors, are often added to cell cultures to facilitate adhesion and enhance cell proliferation and differentiation, thus promoting tissue formation [15]. Growth factors are large biomolecules that consist of smaller proteins that act as signalling molecules for the cell. A common reagent used in cell culture is foetal bovine serum (FBS) derived from the blood of bovine foetuses, which contains bovine serum albumin, numerous adhesion proteins and a cocktail of other components [21]. However, despite its widespread use, there are serious concerns about the use of FBS in clinical applications, due to its high cost, batch reproducibility and issues associated with animal welfare. Therefore, there is currently a drive to reduce the reliance on FBS in tissue engineering through achieving the effect of FBS via scaffold modifications or serum substitutes, Figure 2 [22].

Scaffolds provide the 3D framework and support for seeded cells to attach, spread, proliferate and eventually form into tissue [23]. The porous nature of the scaffold allows for high mass transfer and waste removal [16]. A wide range of scaffolds have been produced from synthetic materials, such as polymers and composites, as well as naturally sourced materials and decellularised human/animal tissue [24,25]. Scaffolds fabricated from natural biomaterials possess the chemical structures that can mimic native tissue, aiding biocompatibility, and can be recognised by the body, however they often lack the requisite mechanical strength and their origin can lead to complications such as premature scaffold degradation, particularly production in large quantities at a commercial scale from limitations due to raw material availability or lot-to-lot (or batch-to-batch) variations [26,27]. In contrast, synthetic materials have well-defined chemical compositions which allows for precise control over mechanical properties and degradation rates, as well as production in almost unlimited quantities [28]. However, these may require addition of growth factors to initiate cell adhesion and may have issues around biocompatibility as they often lack the necessary binding site for cell recognition [29,30]. Hence, the type of scaffold used in culturing tissue is not only paramount for the successful generation of tissue, but can also govern the applications accessible.

Recent advances in cell culture include applications other than regenerative medicine, such as “cellular agriculture”, whereby cells are cultured in a scaffold to form meat fit for human consumption—an alternative to livestock meat production [31]. This emerging application of tissue engineering has potentially beneficial environmental implications, as a more efficient, non-methane producing means to produce meat [32]. Another application of tissue culture is the production of functional tissue analogues used in the pharmaceutical industry for drug screening to reduce this industry’s reliance on vivisection (particularly early in the drug screening process) [33].

### 1.2. Cellulose as a Sustainable Scaffold for Tissue Engineering

Recently, principles of sustainability, eco-efficiency and green chemistry have begun to guide the development of a new generation of materials as an alternative to conventional polymers based on conversion of fossil carbon (e.g., oil) [34,35]. There are a wide range of biomaterials currently used in tissue engineering such as proteins, polysaccharides and biodegradable polymers. Protein and polysaccharide based biomaterials have been reviewed previously as nanoparticle scaffolds for tissue engineering [36]. Biodegradable and biocompatible polymer scaffolds have been reviewed [37] and an overview of hydrogels based on natural polymers and their various applications in the field of tissue engineering was published in 2011 [38]. For completeness, the reader is also referred to the review of decellularised whole-organ scaffolds by Peloso et al. [26], although, as such scaffolds are derived from (deceased) human donors, supply is limited and some of the concerns associated with animal derived scaffolds apply.

**Figure 3:**
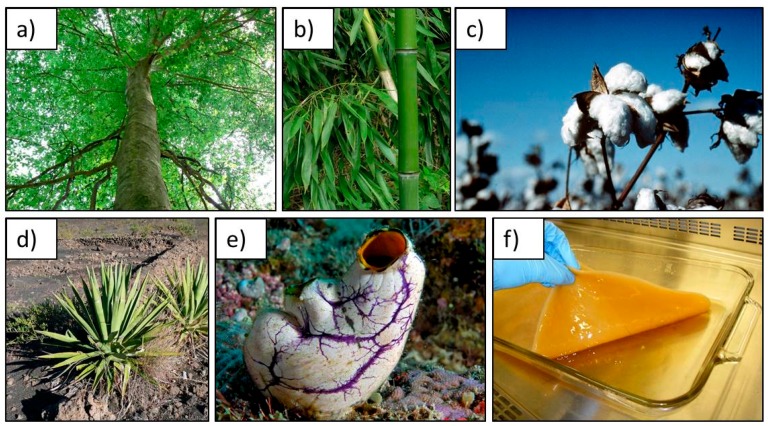
Cellulose can be obtained from various sources: (**a**) beech tree; (**b**) bamboo; (**c**) cotton; (**d**) sisal; (**e**) tunicine; and (**f**) *Gluconacetobacter xylinus*.

The most common natural biomaterials are polymeric in nature and either protein-based, such as collagen, elastin, gelatin and silk, or polysaccharide-based, such as chitosan, alginate, hyaluronic acid and cellulose [39]. One of the most promising of these natural biomaterials, which has received much attention, is the polysaccharide cellulose. Much of this focus on cellulose is due to it possessing the necessary scaffold properties for tissue engineering, such as its biocompatibility, and relative readiness to be tuned through chemical modification to adjust mechanical properties and introduce various surface modifications. In addition, the sustainability of producing and manufacturing materials from cellulose, as well as its modest cost, makes cellulose an economically viable feedstock [40,41,42,43,44]. Cellulose can be sourced from a range of natural materials, most commonly from the cell wall of plants, where it is the major component. Other sources include tunicates and cellulose synthesised by bacteria, such as *Gluconacetobacter xylinum* (Figure 3). As the most abundant biopolymer on the planet [45], cellulose is considered an almost inexhaustible source of raw sustainable material, with an estimated 28.2 billion tonnes produced via biomass annually [40].

Regardless of origin, the chemical structure of cellulose is the same: Anhydroglucose units connected by β-1,4 glycosidic bonds between the C1 and C4 positions [39]. Unlike its monomer, glucose, cellulose is insoluble in water and many organic solvents. The lack of solubility arises due to the presence of intramolecular bonding and strong hydrogen-bonding between cellulose polymer chains, which extend to interfibril interactions. This lack of solubility makes solution processing challenging and, as cellulose is not a thermoplastic material (it does not melt), it cannot be formed using typical melt extrusion techniques. This can result in processing challenges, but recently it has been demonstrated that cellulose dissolved in ionic liquid solutions may be processed into the desired structure and form by: Electrospinning, casting or moulding, before being regenerated in an anti-solvent, such as water and ethanol [46]. The degree of polymerisation (number of monomeric units in the polymer chain, DP) of the cellulose backbone is dependent on where it is sourced from, as well as how it is processed, which, in turn, affects its material properties. For example, bacterial cellulose has a DP of 800–10,000 repeat units, whilst the DP of cellulose from wood pulp is only 300–1700 [40]. Differences in the DP can affect the viscosity of cellulose solutions, as well as the mechanical properties of the final processed product.

There are many different types of cellulose particles that can be obtained (summarised in Table 1), including bacterial cellulose (BNC), microfibrillated cellulose (MFC), nanocrystalline cellulose (NCC), regenerated cellulose and decellularised plant tissue [55]. These have been widely investigated as potential materials for tissue engineering, due to their biocompatibility, biodegradability, and low cytotoxicity as well as tuneable chemical and physical properties [53,56]. Bacterial cellulose is formed as the *Acetobacter* bacterium extrudes pellicles of very pure cellulose fibrils and can be produced sustainably on scale using bioreactors [57]. When growing bacterial cellulose, the pellicles rise to the surface of the reactor and agglomerate, forming a membrane. Due to the high purity of cellulose these can be used as dense hydrogels, processed into nanofibrils, or solubilised or dispersed for further processing into formed materials. Membranes of bacterial cellulose are already used clinically as dressings to treat burn wounds as they have a high water content, do not adhere to healing skin and can be sterilised [58].

Other forms of cellulose nanofibres include MFC fibres, mainly sourced from wood pulp [48]. The wood pulp is delaminated by mechanical pressure before being treated chemically, or enzymatically, to produce nanofibres 5–60 nm wide and several microns long. NCC is produced by treating wood pulp (or other cellulose sources) with concentrated sulfuric acid, to dissolve the non-crystalline domains of the fibres, followed by high pressure homogenisation to fully disintegrate the nanoparticles [59]. These nanocrystals are the smallest type of cellulose particle, have a cross-sectional diameter as low as 5 nm and are 100 s of nm in length, whereas MFC and BNC are several microns in length [55]. Although cellulose is considered to be a highly sustainable material it is important to note that the mechanical disintegration of the wood pulp fibres can be very energy intensive at scale [60]. However, a more environmentally friendly process to produce cellulose nanofibrils (CNF) has been identified. This relies on an oxidative chemical modification using 2,2,6,6-tetramethylpiperidine-1-oxyl (TEMPO) and an oxidant after the acid hydrolysis step, which significantly reduces the energy requirement of the homogenisation process from 20,000—30,000 kWh/tonne to 1000 kWh/tonne [61].

Another key advantage with using cellulose is that it can be processed into an array of materials. Cellulose nanocrystals can be dispersed to form delicate hydrogels [62], cellulose solutions can be electrospun into nanofibres [63] or regenerated as films [44] as well as formed into porous 3D structures [64]. Each of these has different mechanical and physical properties beneficial for specific tissue culture applications. Complex tissue formation requires a level of vascularity in scaffolds to allow mass transfer of nutrients and waste. Some plant tissue has vascular structures similar to human tissue and scaffolds can be prepared by decellularising the plant tissue [65]. This process is a convenient way to obtain complex structures without the need for multiple processing stages [66].

**Scheme 1:**
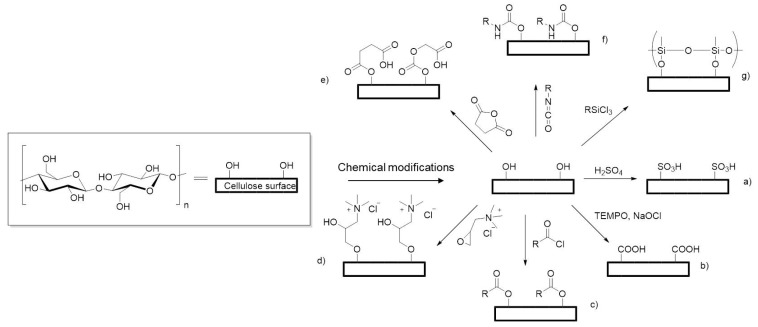
The chemical structure of the anhydroglucose unit in cellulose and examples of some chemical modifications possible by reaction of the hydroxyl groups exposed on the surface of CNF: (a) sulfonation; (b) 2,2,6,6-tetramethylpiperidine-1-oxyl (TEMPO) mediated oxidation; (c) ester formation by reaction with acid chlorides; (d) grafting of tetra-alkylammonium groups by reaction with glycidyltrimethylammonium chloride (GTMAC); (e) grafting via ester linkages generated by reaction with acid anhydrides; (f) formation of urethanes by reaction with isocyanates; and (g) silylation. Reproduced with permission from Courtenay et al., 2018, published by The Royal Society of Chemistry [67].

Not only does cellulose have tuneable mechanical and structural properties, but it also can be readily functionalised due to the exposed hydroxyl groups on the surface of the fibrils, summarised in Scheme 1. Common modifications include the TEMPO oxidation of the hydroxyl group to a carboxylic acid [68], cationisation by grafting of glycidyl trimethylammonium chloride to the surface to introduce a positive charge [67], sulfuric acid hydrolysis leading to sulfate half esters [69] and derivitisation to produce a range of cellulose esters and ethers [70]. Although different modifications of cellulose materials have been widely reviewed [45,71,72] and exploited for other applications such as water purification [73], drug delivery [74] and rheology modification [75], reports of use in tissue engineering applications are more recent. This review focusses specifically on the use of modified cellulose materials for tissue culturing applications, including modifications and tissue culture applications summarised in Table 2.

## 2. Methods of Scaffold Modification

Modifications applied to cellulose materials to be used as tissue scaffolds can be divided into three main categories following trends in the recent literature:

1. Physical modifications—composites and blends;
2. Biochemical modifications—grafting of biomolecules onto the surface;
3. Chemical modifications—introducing new functional groups.

### 2.1. Physical Modifications

Composite scaffolds can be prepared through blending a cellulose powder, dispersion or solution which another material, often a polymer or an inorganic component. The benefits of blending cellulose with other materials are the ability to modulate or introduce new properties beneficial to the application in question, for instance introducing a charge [64], altering topography [104], or varying the mechanical properties [105,106]. This allows the creation of a family of cellulose composites.

Bacterial cellulose offers certain advantages for tissue engineering as it possesses high purity and an ultrafine fibrous network structure with variable porosity. Furthermore, it can be produced into different shapes and moulded into 3D structures during in vitro cultures [107]. Hydroxyapatite is commonly added to cellulose scaffolds as it is biocompatible, increases the tensile properties and promotes calcium phosphate mineralisation, which is valuable for bone tissue generation [108]. Scaffolds with pores in the micrometre and nanometre range have been prepared by blending bacterial cellulose with tri-calcium phosphate and hydroxyapatite and such scaffolds could be used to form implants for bone tissue engineering as mineralisation occurs on the hydroxyapatite [82]. By forming layers of bacterial cellulose, harvested from the floating pellicles at the air-liquid interface, with hydroxyapatite or glycosaminoglycans, a nanocomposite scaffold could be fabricated that was biocompatible and mimicked the nanoscale fibrous structure of bone and cartilage ECM, respectively, resulting in tissue constructs that could regenerate osteochondral defects when implanted into the body [84]. Furthermore, hydrogels have been formed by gelation of bacterial cellulose nanofibres, stabilised by procyanidins, and blended with collagen and hydroxyapatite. Once lyophilised, these scaffolds supported the growth of human bone marrow stromal cells and osteoblastic differentiation was observed after 10 days by detecting the level of alkaline phosphatase expressed [83]. Compared with pure bacterial cellulose, the addition of both gelatin and hydroxyapatite improved the osteoinductivity of the scaffolds, vital for application for the culturing of bone tissue.

Cellulose can also be blended with other polysaccharides such as chitin, chitosan and alginate, to produce novel biomaterials. Chitin and chitosan are similar in structure to cellulose, being comprised of anhydroglucosamine units—*N*-acetylated in the case of chitin. Chitosan is not as robust as cellulose, as it is solubilised in weak acidic solutions, however it does exhibit a slightly positive charge when protonated and will absorb to cellulose surfaces, which are weakly negatively charged in aqueous media [109]. Chitosan-cellulose scaffolds have been developed by regenerating cellulose in an anti-solvent solution of chitosan and used to support MG-63 cell attachment and spreading [93].

Complex 3D scaffolds made from a gel composed of nanocellulose and nanochitin were fabricated using sacrificial templating of a methacrylate and acrylamide resin. Computer aided design enabled a 3D template to be printed with features of ~50 µm, which was filled with the nanocellulose-nanochitin gel. The scaffold template was removed in an alkaline solution leaving a highly porous interconnected biomimetic scaffold, which provided a stiff microenvironment necessary to facilitate the differentiation of human mesenchymal stem cells (hMSCs) [64]. Alginate, in comparison, is an anionic polysaccharide and can be easily fabricated by crosslinking with Ca^2+^ ions [85]. Scaffolds have been produced by mixing bacterial cellulose with alginate hydrogels and directionally freeze-drying to create a composite material with an open porous structure that supports the growth of L929 mouse fibroblast cells [85]. The advantage of blending chitin, chitosan or alginate into the scaffolds is they are all degradable in vivo [110], whereas cellulosic materials are biodurable and are absorbed into the tissue, but can be degraded into glucose in the presence of added cellulase enzymes in vivo [111].

Cellulose is often used as a matrix to support other materials beneficial for cell culture. Pectin is used in tissue engineering as cells can be embedded into the structure, however, it has poor mechanical properties. To overcome this, pectin has been blended with a water soluble cellulose derivative, Carboxymethyl cellulose (CMC) and further reinforced with MFC. Lyophilised CMC/MFC/pectin composite hydrogels have been shown to support viable cells of the NIH3T3 fibroblast cell line [101]. The solubility of CMC in water means that it can be easily mixed with gelatin to form hydrogels. To improve the stability, hydrazide-modified gelatin and aldehyde-modified CMC, which readily crosslink to form stronger hydrogels, have been used. The fabrication of micro-channels in the hydrogel mimic the vascular networks in healthy tissue and cells can be embedded within these channels as a step towards engineering vascularised and cell-dense 3D tissues [100].

Other popular scaffolds produced from cellulose include electrospun nanofibres. This is a relatively simple technique to produce mats of entangled nanofibres with a high surface area, open porous structure and high tensile strength. This method requires the dissolution of cellulose and it is often first converted to cellulose acetate via a mercerisation-acetylation method, to improve its electrospinnability [103]. Once in solution other polymer additives are commonly added; for example poly(lactic acid) (PLA) and poly(dioxanone) (PDO) are both biodegradable polymers which will influence mechanics and degradation rate of the scaffold in vivo [103]. The electrospun mats formed supported L929 mouse fibroblast proliferation and cell infiltration into the scaffold, as well as biomineralisation of nano-hydroxyapatite deposits on the fibres [103].

Cellulose nanocrystals have been added to polymer solutions to reinforce the resultant extruded fibres. A copolymer of maleic anhydride modified poly(butylene adipate-co-terephthalate) was dispersed with cellulose nanocrystals by extruding the copolymer solution into the cellulose dispersion [90]. The addition of cellulose nanocrystals increased the elastic modulus and tensile strength of the fibres, as well as improving the low thermal stability and raising the glass transition temperature, *T_g_*, of the composite. As little as 9% cellulose nanocrystals in the composite significantly enhanced L929 mouse fibroblast cell adhesion [90]. Nanocrystal cellulose has been used as a nanofiller additive, along with reduced graphene oxide, to make thin films of PLA [91]. The presence of cellulose nanocrystals significantly increased the tensile strength of the film up to 23% and improved the ductile properties. The nanocomposite films produced by this method showed antibacterial activity and in vitro cell based cytotoxicity assays confirmed biocompatibility with the fibroblast cell line NIH-3T3.

Fabricating composite scaffolds from blends of cellulose nanofibres can be considered more environmentally friendly than scaffolds made from regenerated cellulose because this removes the need for using ionic liquids in manufacturing. Solubilising cellulose in ionic liquids can add to the processing costs and ionic liquids can be toxic to cells if left in the material, so rigorous cleaning procedures need to be included to ensure that no ionic liquid remains [112]. Nonetheless, ionic liquid aided processing facilitates formation of a range of materials by solution casting and phase inversion methods [113] and use of a range of co-solvents can facilitate co-dissolution of other components [114,115]. Variation of anti-solvents in phase inversion directly impacts on the porous nature of the materials [116].

### 2.2. Biochemical Modifications

Despite having many beneficial properties for a tissue scaffold, one potential limitation of using cellulose is its hydrophilic nature and low non-specific protein binding affinity, which means that mammalian cells do not readily absorb onto cellulose surfaces [117,118,119]. This can be overcome by the introduction of biomolecules, such as matrix ligands, growth factors, or FBS either contained in the cell growth media, or functionalised onto the scaffold surface, to facilitate initial cell attachment [120].

RGD (Arg-Gly-Asp) is commonly used to facilitate cellular adhesion onto scaffolds as it is the minimal fragment of the active site of cell adhesive proteins such as fibronectin [121]. Bacterial cellulose hydrogels have been modified with xyloglucan-RGD conjugates to enhance the attachment and proliferation of human endothelial cells [79]. Carbohydrate-binding modules (CBM) are protein domains present in cellulose-degrading enzymes and have an affinity to cellulose surfaces. These have been used as intermediaries to attach biological molecules, which would not readily bind to native cellulose, onto cellulose surfaces [116]. The recombinant protein IKVAV (Ile-Lys-Val-Ala-Val), is another cell adhesion motif found in the ECM which has been attached onto the surface of a bacterial cellulose scaffold using CBM3, resulting in an appropriate environment for promoting neural and MSC adhesion [81].

To direct the development of vascularised structures, angiogenesis, growth factors such as VEGF are required. However, it is necessary to incorporate VEGF into the scaffold matrix as it has a short half-life and can readily diffuse into the media in vivo [122]. 3D porous scaffolds from bacterial cellulose/gelatin composites were surface modified with heparin, via a condensation reaction, in order to bind VEGF onto the surface through electrostatic interactions between negatively charged *N*- and *O*-sulfated groups of heparin and the basic lysine and arginine residues of VEGF. By fixing VEGF onto the scaffold surfaces, the sustained delivery of VEGF, required to facilitate the production of new blood vessels in the tissue construct, was enabled [80].

The addition of biological molecules onto the scaffold surface can also enhance the biocompatibility of the biomaterial. Biomimetic scaffolds have been produced from electrospun PLA/CNF composite nanofibres coated in a dopamine solution, to form a layer of poly(dopamine) (PDA) on the surface [95]. The addition of PDA onto the surface of the scaffold increases the adhesion of hMSCs, due to the large amount of amine and hydroxyl groups present. CNF have also been electrospun with poly(2-methacryloyloxyethyl phosphorylcholine) before being coated with PDA [102]. This formed a zwitterionic polymer coating, limiting the fouling on the nanofibre membranes necessary for biomaterials for wound healing or tissue engineering, where antibacterial scaffolds are required. However, a disadvantage to the technique is the deposition of dopamine, which is a very time-consuming process, taking up to several days [95].

Along with animal based proteins, there are several types of proteins derived from plants that can be used to enhance the biomimetic nature of the scaffold [123]. In particular, soy protein isolates (SPI) have been grafted onto oxidised cellulose in order to absorb growth factors necessary for in vitro biomineralisation. When the scaffolds were soaked in a doubly concentrated simulated body fluid solution, biomimetic calcium phosphate mineralisation was initiated, producing hydroxyapatite rod-like nanocrystals, a perquisite for bone tissue engineering [89]. However, a disadvantage of using SPI is its solubility in acidic or basic media.

### 2.3. Chemical Modifications

The three primary alcohol groups present in the anhydroglucose unit makes cellulose very amenable to functionalisation as these are exposed at surfaces, e.g., of nanofibrils, sheets, or nanocrystals. This enables new chemical and physical scaffold properties to be introduced or further tuned. Oxidation of CNF is an attractive modification method as it changes the behaviour of the nanofibrils, rendering these readily dispersible in water. This allows for cellulose to be processed in a viscous liquid form without requiring ionic liquid solvents [75]. Surface hydroxyl groups on bacterial CNF were oxidised by TEMPO to carboxylic acid groups and used to disperse hydroxyapatite nanoparticles. Upon addition of gelatin, a hydrogel was formed and crosslinked by glutaraldehyde, producing a scaffold which showed potential for engineering bone tissue [78]. Oxidation using acidified sodium periodate forms dialdehyde cellulose (DAC) and scaffolds have been fabricated by blending DAC with collagen, followed by crosslinking to form a 3D porous sponge that demonstrated dielectric behaviour, indicating a material that could be suitable for neural tissue engineering focused on the regeneration of the nervous system [86].

Cellulose nanocrystals can also be modified to become water dispersible (often described as “water-soluble”, although clearly the crystals are not dissolved). Scaffolds of highly esterified acetate cellulose nanocrystals (ACNC) were prepared through an environmental friendly single step esterification method resulting in materials with a degree of substitution of 2.18, making these hydrophobic, oleophilic and lipophilic. Ice-templating and freeze drying yielded interconnected, highly porous scaffolds, creating a microenvironment suitable for tissue engineering [87].

Furthermore, the chemical modification of microcrystalline cellulose by phosphorylation, using a molten phosphorous acid-urea reaction mixture, resulted in a water soluble material which could be cast into pellets. Normal human dermal fibroblast were viable on the phosphorylated surface, which was said to mimic the glucosaminoglycans of in vivo cartilage tissue [88]. Cellulose phosphate is more hydrophilic than native cellulose, but it is beneficial for bone tissue generation as calcium can easily be mineralised [124].

Electrospun scaffolds are often used due to their good tensile mechanical strength and as mimics of the fibrous structure of the ECM. However, native cellulose is a poor candidate for electrospinning due to its poor solubility on most organic solvents. Cellulose is often converted to cellulose acetate to be electrospun into fibres. Further modifications have been applied to the nanofibres including oxidation followed by sulfonation to form water-stable sulfated cellulose [94]. These fibrous meshes have demonstrated potential for bone tissue engineering as the sulfate groups are able to retain the osteogenic growth factor, human recombinant bone morphogenetic protein-2 (rhBMP-2), which supports the growth of bone marrow stromal cells on a bone tissue scaffold [94].

Attempts have been made to utilise the existing structure of plants for tissue engineering. Given that cellulose is a major component of the plant cell wall, plant tissues can be decellularised and used as scaffolds. The mechanical structure of cellulose can be modified using chemical cross-linkers such as glyoxal or glutaraldehyde to stiffen the scaffold [125]. Scaffolds derived from apple hypanthium tissue were decellularised, coated with collagen and crosslinked with glutaraldehyde to stiffen the material [66]. Apple tissue was used as a promising candidate for in vitro culture of mammalian cells in a 3D environment because its internal structure consists of connected pores and air pockets needed to transfer nutrients and waste produced in 3D tissue [66]. These scaffolds supported a range of cell lines as well as being easily produced, inexpensive and originating from a renewable, sustainable source.

It has been reported that the contractility of fibroblast cells on a native bacterial cellulose scaffold surface is much lower than between other fibroblast cells [126]. This is detrimental for cell attachment and proliferation on these scaffolds, as the seeded cells would tend to round up instead of elongate [126]. Bacterial cellulose has been functionalised with organosilanes, by grafting methyl terminated octadecyltrichlorosilane or amine terminated 3-aminopropyltriethoxysilane [77]. These modifications increased both hydrophobic and electrostatic interactions with fibroblast cells, beneficial to promote cell growth for wound dressing applications [77]. The growth of fibroblast cells was enhanced on the mannosylated surface of bacterial cellulose membranes, achieved by grafting aryl monosaccharides into succinylated bacterial cellulose [76]. The covalent attachment of carbohydrates onto the surface was made viable through the succinic crosslink and was reported to be beneficial to the stimulation of fibroblast growth, as it is a monosaccharide motif used in cellular recognition. Furthermore, the cells have a higher affinity for the succinylated bacterial cellulose due to a higher charge on the carboxylated surface.

**Figure 4:**
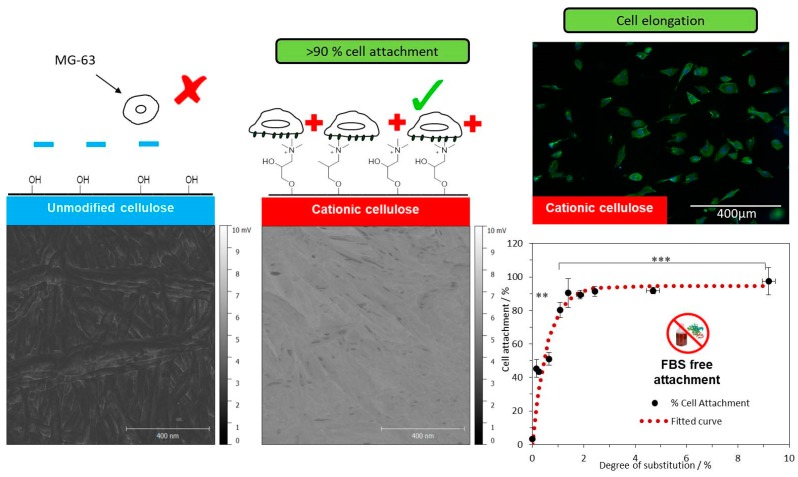
Cationisation of cellulose film by GTMAC introduced a positive charge to the surface facilitating MG-63 cell attachment, in a matrix ligand free system, whereas only minimal attachment was observed on unmodified cellulose surfaces. Electric force microscopy revealed the cationic cellulose had a positive surface charge compared to the negatively charged native cellulose. Interestingly only a low level of ~1.4% degree of substitution was required to induce this effect. After 24 h incubation at 37 °C in 5% CO_2_ greater cell elongation occurred in on the cationic scaffolds compared to the unmodified cellulose. Reproduced with permission from Courtenay et al., 2017 [92], Copyright © 2016, Springer Nature.

Cellulose surfaces bear a slight negative charge in aqueous media [109] and, to overcome this, the epoxide, glycidyltrimethylammonium chloride (GTMAC), has been used to introduce a positive charge onto the surface via introduction of quaternary ammonium moieties. Unlike chitosan, these cationised CNF have a permanent charge and can be dispersed in water to form stable hydrogels if the degree of GTMAC substitution along the nanofibril is high enough to charge stabilise the dispersed particles [67]. Bacterial cellulose films were chemically modified with GTMAC facilitating the attachment of MG-63 osteoblast cells through electrostatic interactions between the phosphate-lipid bilayer of the cell membrane and the positively charged quaternary ammonium group (Figure 4 [22]). Importantly, this was achieved in the absence of matrix ligands needed for cell attachment—no FBS was present in the culture medium during attachment. Reducing the reliance on growth factors, or proteins, for cell culture is important for industrial application as these are very costly, can exhibit batch variability and are derived from animals or humans. In addition to modification of the surface charge, the structural properties of the scaffolds could be tuned by crosslinking with glyoxal to increase stiffness and further regulate cellular response [92]. These modifications use simple yet robust chemistries that can be applied to any form of cellulose and are amenable to scaling up for industrial applications.

Other derivatives of cellulose have been chemically modified to produce novel scaffolds and are often used as biomaterials due to their solubility in water and most organic solvents, low cost and commercial availability in a range of molecular weights [99]. HPC was photo crosslinked with methyl methacrylate to form a photo-patterned and biodegradable hybrid paper substrate which could be used for cell culture. Using lithographic techniques enables patterns of modified cellulose to be produced, which could be used to form arrays of discrete cell clusters for cell assays such as toxicity or population dynamics [97]. EHEC and hydrophobically modified EHEC were blended with PVA and electrospun into fibres. These fibres were cross-linked with citric acid and supported the growth of L929 mouse fibroblast cells, showing potential for cell culture applications [98]. Soft, interconnected microporous scaffolds were prepared by modifying HPC with methacrylic anhydride, improving the biodegradability [99]. These 3D hydrogel scaffolds were used to culture human adipose-derived stem cells (ASCs) due to the interconnecting pores aiding nutrient transfer [99].

### 2.4. Cellulose Bioresorbability and Biodegradability In Vivo

An important factor when considering cellulose as a scaffold material for tissue engineering applications is its biodegradation in vivo. Cellulose is commonly referred to as biodegradable, as it is degraded by microorganisms, however the resorption of cellulose in vivo does not occur as animals and humans do not synthesise cellulases [74]. A long-term study by Martson et al. [127], described cellulose-based implants as biodurable as cellulose sponge scaffolds only underwent very slow degradation in rat subcutaneous tissue after 60 days [127]. Whilst this may be a potential limitation for the success of cellulose scaffolds to be used for in vivo tissue engineering; it is not the case for in vitro culture or cellular agriculture applications.

Regenerated cellulose fibres when treated with N_2_O_4_ to produce oxidised cellulose have been shown to bioresorb in vivo [128,129]. Several Johnson and Johnson Medical Inc. patents exist, covering the preparation and use of oxidised cellulose for use as surgical haemostats and gauzes (SURGICEL^®^) to prevent post-operative adhesions [130,131]. Periodate oxidation to introduce aldehyde groups on the cellulose chain has been shown to promote degradation of the cellulose at physiological pH [42,74,132]. However limited advancements have been made since. Another more recent approach involved dosing cellulosic scaffolds with cellulase prior to implantation to stimulate in vivo degradation [42,111]. Moreover, including hyaluronic acid into cellulose structures introduces area of the scaffold that are enzyme degradable [42]. Sannino et al. demonstrated that carbodiimide could be used as a crosslinker between hyaluronic acid and cellulose derivatives [133]. This process introduces ester bonds amongst the cellulose networks, which can be digested via hydrolysis [42,133]. Furthermore, cellulose can be functionalised with several biomolecules, through carbodiimide crosslinking, such as cell function promoting polypeptides [134,135]. Despite limited progress in the last decade to make cellulose degrade in vivo, this has not deterred the breadth of recent literature investigating modified cellulose scaffolds for cell culture.

## 3. Conclusions

It is clearly apparent that there is much potential for cellulose based materials as scaffolds in tissue engineering. These are attractive from both a sustainability point of view as well as industrial applications as there are a range of readily accessed fabrication methods possible. Cellulose is a cost effective and sustainably source biomaterial amenable to an array of modifications that unlock new properties and applications. Whilst there are a vast range of chemistries at hand that can be applied to cellulose, only those that are robust, scalable and amenable to manufacturing are likely to have longevity in tissue engineering beyond the laboratory.

It is very beneficial for the scaffold modifications, either chemical or biochemical, to reduce the reliance of matrix ligands for cell attachment, which is important as currently the majority of matrix ligands are provided by foetal bovine serum, which is not a sustainable source for industrial scale due to high cost, batch variation and ethical considerations arising from to its origin. Moreover, developing scaffolds with complex vascular-like structural features will be important for transitioning cell culture from simple constructs to functional tissues. Sourcing cellulose from decellularised plant tissue can reduce the cost and complexity of processing of the scaffold whilst introducing vascularity onto the scaffold. Furthermore, there are many opportunities to blend cellulose with other biomaterials to obtain a scaffold with the desired properties for specific applications.

Thus, modified cellulose meets the demand for a new biomaterial with suitable properties for tissue engineering: Derived from a sustainable source and requiring minimal chemical processing, or added growth factors, to culture cells for industrial applications.

## Figures and Tables

**Table 1:** A summary of the different types of nanocellulose, origin, formation and sizes. The table was reproduced from Klemm et al., 2011 [47]. Copyright © 2011 WILEY-VCH Verlag GmbH & Co. KGaA, Weinheim.

Type of Nanocellulose	Selected References and Synonyms	Typical Sources	Formation and Average Size
**Microfibrillated cellulose (MFC)**	Microfibrillated cellulose [48], nanofibrils and microfibrils [35], nanofibrillated cellulose [49]	Wood, sugar beet, potato tuber, hemp, flax delamination	Delamination of wood pulp by mechanical pressure before and/or after chemical or enzymatic treatment Diameter: 5–60 nm Length: several micrometres
**Nanocrystalline cellulose (NCC)**	Cellulose nanocrystals, crystallites [50], whiskers [51], rod-like cellulose microcrystals [52]	Wood, cotton, hemp, flax, wheat straw, mulberry bark, ramie, Avicel, tunicin, cellulose from algae and bacteria	Acid hydrolysis of cellulose from many sources Diameter: 5–70 nm Length: 100–250 nm (from plant celluloses); 100 nm to several micrometres (from celluloses of tunicates, algae, bacteria)
**Bacterial nanocellulose (BNC)**	Bacterial cellulose [40], microbial cellulose [53], biocellulose [54]	Low-molecular-weight sugars and alcohols	Bacterial synthesis Diameter: 20–100 nm; different types of nanofiber networks

**Table 2:** A summary of the recent literature on modified cellulose for tissue culture applications.

Cellulose Type	Modification	Scaffold Form	Tissue Culture Application
**Bacterial Cellulose**	Mannosylated	Membranes	Enhanced fibroblast growth [76]
	Cationisation and oxidation	Membranes	Protein free cell attachment [76]
	Silanisation	Lyophilised membranes	Wound dressing [77]
	TEMPO-mediated oxidation	Hydrogel with hydroxyapatite and crosslinked by glutaraldehyde	Bone tissue [78]
	RGD and xyloglucan-peptide grafting	Membranes	Engineering blood vessels [79]
	Modified with heparin	3D porous scaffold loaded with vascular endothelial growth factor (VEGF)	Tissue regeneration [80]
	Peptides fused to a carbohydrate-binding module (CBM3)	Membranes	Promoting neuronal and mesenchymal stem cell (MSC) adhesion [81]
	Tri-calcium phosphate and hydroxyapatite blend	Hydrogel	Bone tissue implants [82]
	Collagen and hydroxyapatite blend	Hydrogel crosslinked by procyanidins	Bone tissue [83]
	Hydroxyapatite and glycosaminoglycan blends	Layered scaffolds	Repair of osteochondral defects [84]
	Alginate blend	Porous scaffold crosslinked with Ca^2+^	Biocompatibility and porous [85]
**Nanocrystalline Cellulose**	Dialdehyde cellulose crosslinked with collagen	3D porous scaffold	Dielectric behaviour relevant to neural tissue engineering [86]
	Acetate esterification	Interconnected highly porous scaffold	Hydrophobic and lipophilic scaffolds [87]
	Phosphorylation	Thin films	In vitro cell culture and in vivo tissue regeneration [88]
	Oxidised cellulose grafted with soybean protein isolate	Scaffold soaked in doubly concentrated simulated body fluid	Biomimetic calcium phosphate mineralisation [89]
	Copolymer dispersed with cellulose nanocrystals	3D nanocomposites	Biomedical and tissue engineering applications [90]
	CNC and reduced graphene oxide blended in PLA matrix	Nanocomposite film	Antibacterial activity [91]
	Nanocellulose blended with nanochitin	CAD generated porous structure	Biomimetic tissue engineering [64]
**Microfibrillated Cellulose**	Cationisation and glyoxalation	Regenerated modified cellulose films	Tailoring scaffold properties to regulate cell response [92]
	Cellulose-chitosan infusions	Hydrogels	Cell attachment [93]
	Oxidation followed by sulfonation	Electrospun fibre meshes	Bone tissue [94]
	Decellularisation followed by glutaraldehyde crosslinking	3D cellulose scaffolds	In vitro culture of mammalian cells in a 3D environment [66]
	Dopamine coated	Electrospun PLA/CNF composite nanofibres	Enhance cell biocompatibility [95]
	Polyurethane coated in a CNF dispersion	Electrospun nanofibres	Tissue engineering [96]
**Cellulose Derivatives**	Hydroxypropyl cellulose (HPC) crosslinked by methyl acrylate	Biocompatible and hydrolytically degradable scaffold	Long term cell culture [97]
	Ethyl hydroxyethyl cellulose (EHEC) crosslinked with citric acid	Electrospun nanofibres	Drug delivery and as scaffolds in tissue engineering [98]
	HPC modified with methacrylic anhydride	3D hydrogel constructed with interconnecting pores	Adipose tissue [99]
	Crosslinked gelatin/carboxymethyl cellulose (CMC) blend	Hydrogel with perfusable vascular networks	Engineering vascularised and cell-dense 3D tissues and organs [100]
	CMC/MFC/pectin blend	Lyophilised hydrogels	Biocompatible composite scaffolds [101]
	Cellulose acetate with polymer graft and polydopamine (PDA) coating	Electrospun nanofibre mats	Antifouling surface [102]
	Cellulose acetate blended with PLA or PDO	Electrospun nanofibre mats	Biomineralisation [103]
